# MicroRNA-101 inhibits the expression of Rhes, a striatal-enriched small G-protein, at the post-transcriptional level in vitro

**DOI:** 10.1186/s13104-018-3654-5

**Published:** 2018-07-31

**Authors:** Hideya Mizuno, Ayako Taketomi

**Affiliations:** School of Pharmacy and Pharmaceutical Sciences, Mukogawa Women’s University, 11-68 Koshien Kyuban-cho, Nishinomiya, Hyogo 663-8179 Japan

**Keywords:** Rhes, microRNA-101, HEK293 cells, Striatum

## Abstract

**Objective:**

Ras homolog enriched in striatum (Rhes) is a small GTP-binding protein that is predominantly localized in the striatal region of the brain. Rhes affects various signaling pathways and plays important roles in Huntington’s disease development caused by striatal anomalies. However, the mechanism underlying the regulation of Rhes expression is not fully understood. We hypothesized that Rhes expression might be regulated by microRNAs (miRNAs), which are small noncoding RNAs that regulate gene expression by interacting with the 3′-untranslated region (3′UTR) of mRNA. This study therefore investigated the interaction between miRNAs and the Rhes mRNA 3′UTR.

**Results:**

The results of luciferase assay showed that miR-101, the miRNA determined to have the highest possibility of interacting with the Rhes mRNA 3′UTR using DIANA-microT, significantly inhibits luciferase activity, suggesting that miR-101 directly targets the Rhes mRNA 3′UTR. Additionally, Rhes protein levels in cultured cells co-transfected with a plasmid containing the complete Rhes cDNA and miR-101 were significantly downregulated by miR-101 as demonstrated by western blot analysis. These results support our hypothesis that Rhes expression is regulated by miRNA and indicate that miR-101 may be a potent modulator of Rhes expression in striatal neurons.

**Electronic supplementary material:**

The online version of this article (10.1186/s13104-018-3654-5) contains supplementary material, which is available to authorized users.

## Introduction

Ras homolog enriched in striatum (Rhes) is a small GTP-binding protein predominantly localized in the striatal region of the brain. Despite being a Ras family member, overexpression of the wild-type or mutant of Rhes does not induce cellular transformation [1]. Rhes affects dopamine-mediated signaling and behavior [2–5] and regulates the PI3K-AKT pathway [6–8]. Notably, Rhes plays important roles in the development of specific diseases caused by striatal anomalies. Subramaniam et al. [9] implicated Rhes in the pathology of Huntington’s disease (HD), which is characterized by striatal neuronal death and is caused by mutant Huntingtin (HTT) containing an expansion of glutamine residues. In their study, Rhes bound more strongly to mutant HTT than to wild-type HTT and induced cytotoxicity in in vivo and in vitro HD models. Furthermore, Rhes affects mammalian target of rapamycin (mTOR) signaling activation in the striatum [10]. Since mTOR signaling is implicated in diverse biological activities that are critical for cell survival and function [11], altered expression of Rhes might affect the cellular condition. Therefore, it is important to elucidate the regulatory mechanism underlying Rhes expression, which is not fully understood.

In order to study the mechanism, we hypothesized that Rhes expression might be regulated by microRNAs (miRNAs). miRNAs are small, approximately 22 nucleotide-long noncoding RNAs that interact with the 3′-untranslated region (3′UTR) of mRNA and physiologically regulate gene expression at the transcriptional and post-transcriptional levels [12]. The expression levels of specific miRNAs are altered in various diseases [13]. Because miRNA expression is altered by epigenetic changes and environmental factors, miRNAs may alter protein expression levels and influence disease onset. Therefore, it is possible that miRNA-associated changes in Rhes expression are involved in the onset of diseases such as HD.

In order to investigate the hypothesis, we examined the expression of Rhes with synthesized miRNAs in cell culture systems using HEK293 cells transfected with each construct by liposome-mediated transfection.

## Main text

### Materials and methods

#### Plasmids, miRNA mimics, and miRNA inhibitors

Full-length cDNA of human Rhes (pOTB7-RASD2) was provided by RIKEN BRC through the National Bio-Resource Project of MEXT, Japan. For its expression in cultured cells, Rhes cDNA was inserted into pcDNA3.1 vector (Invitrogen; Carlsbad, CA, USA). For the luciferase assay, the part of the 3′UTR (1731–1770) in the human Rhes gene containing the expected miR-101 binding site was cloned into pmirGLO Dual-Luciferase miRNA Target Expression Vector (Promega; Madison, WI, USA). The sequences in pmirGLO-Rhes were modified using a PrimeSTAR Mutagenesis Basal Kit (Takara Bio; Shiga, Japan) according to the manufacturer’s instructions. microRNA mimics (double-stranded synthesized mature microRNA), hsa-miR-101, hsa-miR-132, hsa-miR-124 and miR-sc (scrambled) as a negative control were purchased from B-Bridge (Tokyo, Japan).

#### Cell culture and transfection

HEK293 cells (provided by RIKEN BRC through the National Bio-Resource Project of MEXT, Japan) were cultured in Dulbecco’s modified Eagle’s medium (DMEM; Nacalai Tesque; Kyoto, Japan) supplemented with 10% fetal bovine serum (FBS; Biowest; Nuaille, France) in air containing 5% CO_2_ at 37 °C. SH-SY5Y cells (provided by ATCC; Manassas, VA, USA) were cultured in DMEM/Ham’s F-12 medium with 10% FBS in air containing 5% CO_2_ at 37 °C. The cells were transfected with each construct using Lipofectamine 2000 (Invitrogen) or NeuroMag (Oz Biosciences; San Diego, CA, USA) at 1 day after plating (70–80% confluency) according to the manufacturer’s instructions.

#### Luciferase reporter assay

Luciferase activity of lysates of cells transfected with the reporter vector was measured using the Dual-Glo Luciferase Assay System (Promega). Briefly, HEK293 cells were co-transfected with miRNA mimics and pmirGLO-Rhes or pmirGLO (as a control) in a 96-well plate. At 24 h after transfection, the Dual-Glo Reagent was added to each well containing transfected cells and mixed well. After 10 min, luminescence from firefly luciferase was measured using a Tristar LB 941 Multimode Microplate Reader (Berthold Technologies; Bad Wildbad, Germany). Dual-Glo Stop & Glo Reagent was then added to each well, and luminescence from Renilla luciferase was measured after 10 min. Firefly luciferase activity was normalized to Renilla luciferase activity, and this ratio was then normalized to that of a control well containing cells transfected with negative miRNA mimics. The relative activities were finally calculated from the normalized ratios.

#### Western blot analysis

Cells were disrupted in cell lysis buffer (20 mM Tris pH 8.0, 150 mM NaCl, 1 mM EDTA, 1% Nonidet P-40, 0.1% Triton, 50 mM NaF) containing a protease inhibitor cocktail (Nacalai Tesque). SDS-PAGE and western blotting were performed according to general procedures. Anti-Rhes and anti-actin clone C4 (Millipore; Burlington, MA, USA) were used as primary antibodies, and horseradish peroxidase (HRP)-conjugated anti-mouse and anti-rabbit IgG were used as secondary antibodies (Jackson Immuno Research; West Grove, PA, USA). The protein bands on the membrane were detected by a chemiluminescence method using an Immobilon™ Western Chemiluminescent HRP substrate (Millipore) or Amersham™ ECL™ Prime Western Blotting Detection Reagent (GE Healthcare; Buckinghamshire, UK) and analyzed using the ImageQuant™ LAS 4000 biomolecular imager (GE Healthcare).

#### RNA isolation and quantification of mRNA and miRNA

Total RNA, including miRNA, was extracted from cultured cells using the mirVana miRNA isolation kit (Ambion, Austin, TX, USA) according to the manufacturer’s instructions. For mRNA quantification, total RNA was reverse transcribed using a High-Capacity cDNA Reverse Transcription Kit (Life Technologies; Gaithersburg, MD, USA). The real-time polymerase chain reaction (PCR) with specific primers and SYBR green dye was carried out on an Applied Biosystems™ 7000 Real-Time PCR System (Life Technologies). The forward (F) and reverse (R) primer sequences were as follows: Rhes 5′-CAGTGTGCCCGCCAAAAC-3′ (F), 5′-TGGGTGTGTACTGGTCCTCAA-3′ (R); Ribosomal protein L32 (RiboL32) 5′-GAAACTGGCGGAAACCCA-3′ (F), 5′-TGGTGATCCTCTTGTAGCTCTCC-3′ (R). The PCR conditions were 50 °C for 2 min, 95 °C for 10 min, followed by 40 cycles of 95 °C for 15 s, and 60 °C for 1 min.

For miRNA quantification, total RNA was reverse transcribed using the TaqMan miRNA Reverse Transcription kit (Life Technologies) and miRNA-specific stem-loop primers (part of TaqMan miRNA assay kit; Life Technologies). The miRNA expression levels were quantified by real-time PCR using individual miRNA-specific primers (part of TaqMan miRNA assay kit) on the Applied Biosystems™ 7000 Real-Time PCR System according to the manufacturer’s instructions.

#### Statistical analysis

The data are expressed as mean values ± SEM. Statistical analysis was performed with one-way ANOVA followed by Tukey’s multiple comparison test or Student’s *t*-test using Prism software (GraphPad Software; San Diego, CA, USA). The significance level was set at p < 0.05.

### Results and discussion

#### Rhes mRNA 3′UTR is a target of miR-101

**Fig. 1:**
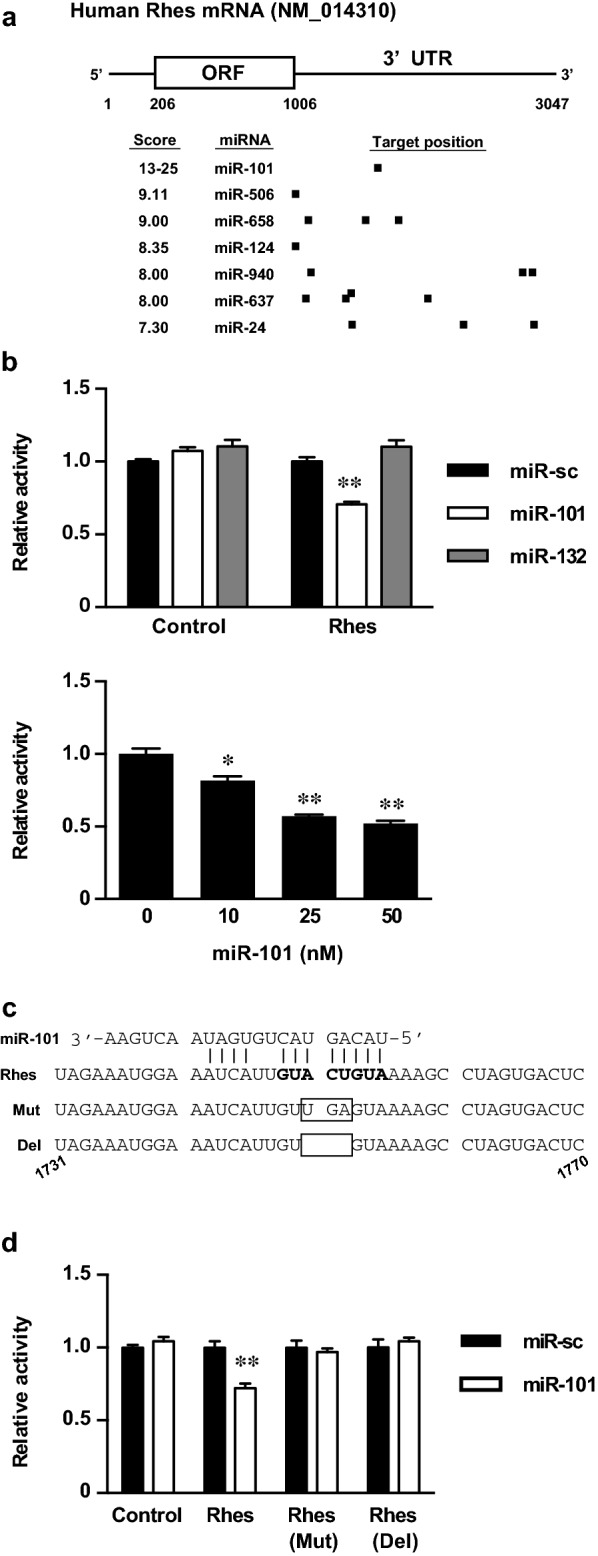
miR-101 targets Rhes mRNA 3′UTR. **a** Scheme of Rhes mRNA structure and miRNAs that bind to Rhes mRNA 3′UTR as predicted by DIANA-microT. **b** Luciferase assay using a part of the Rhes mRNA 3′UTR and miRNAs. (Upper) Luciferase activity was inhibited by miR-101. HEK293 cells were co-transfected with a reporter vector without the insert (Control) or with Rhes 3′UTR (Rhes) and 50 nM miRNA mimics (miR-sc, miR-101, or miR-132). At 24 h after transfection, the cells were lysed, and the luciferase activity of the cell lysates was measured. The activity was normalized to that of a control transfected with miR-sc. Data are presented as the mean ± SEM, n = 5. (Lower) miR-101 inhibited luciferase activity in a dose-dependent manner. HEK293 cells were co-transfected with a reporter vector containing Rhes mRNA 3′UTR and the miR-101 mimic at specified concentrations. Data are presented as the mean ± SEM, n = 5. **c** Sequence of the part of the Rhes mRNA 3′UTR (1731–1770) containing the predicted binding site for miR-101. Mut, substitution mutant of Rhes mRNA 3′UTR; Del, deletion mutant of Rhes mRNA 3′UTR. **d** The mutant form of Rhes mRNA 3′UTR eliminated the luciferase activity inhibition by miR-101. HEK293 cells were co-transfected with a reporter vector and 50 nM miRNA mimics. The activity was normalized to that of a control transfected with miR-sc. Data are presented as the mean ± SEM, n = 5. *p < 0.05, **p < 0.01

Rhes mRNA transcribed from RASD2 has a 3′UTR of almost 2000 bases (Fig. 1a). We used DIANA-microT to predict the miRNAs that possibly interact with Rhes mRNA and obtained miR-101 as the ranked candidate, which was also predicted by other programs (PicTar and TargetScan) (Fig. 1a and Additional file 1) [14]. miR-101 is expressed ubiquitously, and its dysregulation is reportedly involved in some types of cancer [15–17]. In the brain, miR-101 is involved in Alzheimer’s disease [18, 19], schizophrenia [20], and spinocerebellar ataxia type 1 (a polyglutamine disease caused by the expansion of CAG repeats in ataxin1) [21]. Because miR-101 is expressed abundantly in the brain, it may interact with Rhes mRNA whose expression is limited to the striatum under physiological conditions.

Therefore, we prepared a plasmid construct containing part of the Rhes mRNA 3′UTR with the putative binding sites for miR-101 and investigated its interaction with miR-101 by a luciferase assay using HEK293 cells, which are widely used for reporter gene assays because they show high efficiency of gene transduction. Luciferase activity was significantly inhibited by miR-101 but not by miR-132 or miR-124, which are abundant in neurons (miR-132: Fig. 1b, upper; miR-124: Additional file 2). Furthermore, miR-101 inhibited luciferase activity in a dose-dependent manner (Fig. 1b, lower). We then mutated the seed match sequence in the Rhes mRNA 3′UTR as indicated in Fig. 1c to exclude off-target effects and observed elimination of luciferase activity inhibition by miR-101 (Fig. 1d). These results indicated that miR-101 directly targets the Rhes mRNA 3′UTR.

#### miR-101 inhibits Rhes expression in vitro

**Fig. 2:**
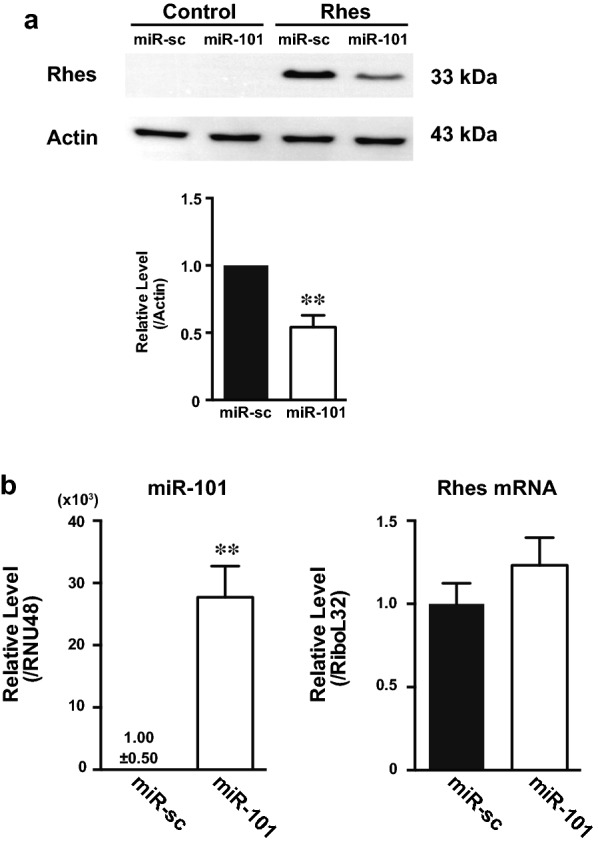
Effect of miR-101 on Rhes expression. Proteins or total RNAs were extracted from HEK293 cells or SH-SY5Y cells transfected with Rhes expression vector and 50 nM miRNA mimics (miR-sc or miR-101). The cells were harvested at 24 h after transfection. **a** Western blot analysis of Rhes expression in HEK293 cells. The immunoblots were normalized to actin expression. Data are presented as the mean ± SEM, n = 6. **b** Quantitative PCR analysis of miR-101 and Rhes mRNA expression in SH-SY5Y cells. Expression was quantified by real-time PCR and normalized to RNU48 or RiboL32 levels. Data are presented as the mean ± SEM, n = 3. **p < 0.01

We investigated the effects of miR-101 on Rhes expression in cultured cells by western blotting. miR-101 was found to significantly downregulate Rhes protein levels (Fig. 2a). We then analyzed endogenous Rhes expression in human neuroblastoma SH-SY5Y cells after introduction of miR-101 (Fig. 2b). Overexpression of miR-101 was not accompanied by altered Rhes mRNA expression, suggesting that miR-101 inhibits Rhes expression at the post-transcriptional level without affecting its mRNA. Then, we tried to investigate the endogenous Rhes protein expression in these cells but could not detect it in our system (data not shown).

## Conclusion

In this study, we indicated that miR-101 inhibits Rhes expression at the post-transcriptional level in vitro. These results support our hypothesis that Rhes expression is regulated by miRNAs and suggest that miR-101 may be a potent modulator of Rhes expression in striatal neurons.

## Limitations

We were not able to detect endogenous Rhes expression in cultured cells with our system. Thus, we could not show any results with the endogenous levels of Rhes and miR-101. However, our results indicated that miR-101 directly targets the Rhes mRNA 3′UTR and inhibits exogeneous Rhes expression. An in vivo animal model may provide further information regarding the role of miR-101 in Rhes expression under normal physiological and pathological conditions.

## Additional files

**Additional file 1.** Predicted miRNAs that bind to Rhes mRNA 3′UTR by using DIANA-microT.

**Additional file 2.** Luciferase activity was not inhibited by miR-124. Luciferase assay using a portion of the Rhes mRNA 3′UTR and miRNAs. HEK293 cells were co-transfected with a reporter vector without the insert (Control) or with Rhes 3′UTR (Rhes) and 50 nM miRNA mimics (miR-sc or miR-124). At 24 h after transfection, the cells were lysed, and the luciferase activity of the cell lysates was measured. The activity was normalized to that of a control transfected with miR-sc. Data are presented as the mean ± SEM, n = 5.

## Abbreviations

3′UTR: 3′-untranslated region
cDNA: complementary DNA
DMEM: Dulbecco’s modified Eagle’s medium
FBS: fetal bovine serum
HD: Huntington’s disease
HTT: Huntingtin
miRNA: microRNA
miR-101: miRNA-101
mTOR: mammalian target of rapamycin
PCR: polymerase chain reaction
Rhes: Ras homolog enriched in striatum
