# Supplementary material for: MicroRNA-101 inhibits the expression of Rhes, a striatal-enriched small G-protein, at the post-transcriptional level in vitro
Source: BMC Res Notes. 2018 Jul 31;11:528. doi: 10.1186/s13104-018-3654-5 (PMC6069827; doi:10.1186/s13104-018-3654-5)
Supplement: Supplementary file 1 — Additional file 1. Predicted miRNAs that bind to Rhes mRNA 3′UTR by using DIANA-microT. [file 13104_2018_3654_MOESM1_ESM.pdf]

DIANA LAB DNA Intelligent Analysis

HOME SOFTWARE DATABASES MEMBERS PUBLICATIONS HELP

DIANA - microT v3.0

How to cite:

1/ M. Maragkakis; P. Alexiou; G. L. Papadopoulos; M. Reczko; T. Dalamagas; G. Giannopoulos; G. Goumas; E. Koukis; K. Kourtis; V. A. Simossis; P. Sethupathy; T. Vergoulis; N. Koziris; T. Sellis; P. Tsanakas; A. G. Hatzigeorgiou. **Accurate microRNA target prediction correlates with protein repression levels.** *BMC Bioinformatics 2009, 10:295* view

2/ M. Maragkakis; M. Reczko; V. A. Simossis; P. Alexiou; G. L. Papadopoulos; T. Dalamagas; G. Giannopoulos; G. Goumas; E. Koukis; K. Kourtis; T. Vergoulis; N. Koziris; T. Sellis; P. Tsanakas; A. G. Hatzigeorgiou. **DIANA-microT web server: elucidating microRNA functions through target prediction.** *Nucleic Acids Research 2009 Jul 1; 37(Web Server issue):W273-6.* view

For issues regarding this application contact **maragkakis[at]fleming.gr**

MORE RECENT VERSION OF MICROT AVAILABLE!

DIANA-microT-CDS, the most recent version of microT algorithm, using data from Ensembl version 69 and miRBase version 18, currently hosts miRNA target predictions for Homo sapiens, Mus musculus, Drosophila melanogaster and Caenorhabditis elegans. The **new and improved web interface** can be accessed through here.

Download all microT v3.0 results

Results for Gene 'ENSG00000100302' (7 miRNAs found) | **New Search**

**Ensembl Gene ID:**	ENSG00000100302
**Gene name:**	RASD2
**Refseq ID:**	NM_014310
**Description:**	GTP-binding protein Rhes precursor (Ras homolog enriched in striatum) (Tumor endothelial marker 2). [Source:Uniprot/SWISSPROT;Acc:Q96D21]
**KEGG pathways:**	
**Species:**	human
**Chromosome:**	22

Score Threshold 7.3

Rank	miRNA name	miTG score	Precision	SNR

1	hsa-miR-101	13.25	0.63	4.64

PubMed Links: gene | miRNA | both

Binding Type	UTR position	Score	Conservation	Also predicted by
8mer(pos 1)	720 - 748	4	5	pictar<br>targetscan

Position on Chromosome 22: 34278746 - 34278774

Conservation Info rn4, mm8, bosTau2, canFam2, loxAfr1

```
3'UTR:  5' ______A_____U________ 3'
           _______AUCAU_GUACUGUA
           _______||||._||||||||
           _______UAGUG_CAUGACAU
miRNA:  3' AAGUCAA_____U________ 5'
```

2	hsa-miR-506	9.11	0.47	3.51

PubMed Links: gene | miRNA | both

Binding Type	UTR position	Score	Conservation	Also predicted by
7mer(pos 2)	8 - 36	5	5	

Position on Chromosome 22: 34278034 - 34278062

Conservation Info rn4, mm8, dasNov1, monDom4, xenTro1

```
3'UTR:  5  _______G__GC__GGCUUGGCCA_______C 3
           ___UGCU_GG__GG__________GUGCCUU_
           ___|.||_.|__||__________|||||||_
           ___AUGA_UC__CC__________CACGGAA_
miRNA:  3' AG_____G__UU___________________U 5'
```

3	hsa-miR-658	9.00	0.8	4.12

PubMed Links: gene | miRNA | both

Binding Type	UTR position	Score	Conservation	Also predicted by
7mer(pos 2)	122 - 150	1	0	

Position on Chromosome 22: 34278148 - 34278176

Conservation Info Not Conserved

```
3'UTR:  5' G___CC__________GG_______A 3'
           _GCC__CGGGC__GCU__CCUCCGC_

           _.||__|||.|__.||__|||||||_
           _UGG__GCCUG__UGA__GGAGGCG_
miRNA:  3' ____UU_____GA___AG_______G 5'
```

Binding Type	UTR position	Score	Conservation	Also predicted by
9mer(pos 1)	643 - 671	1	0	

Position on Chromosome 22: 34278669 - 34278697

Conservation Info Not Conserved

```
3'UTR:  5' A___UCCUCU____GG__________ 3'
           _GCC______CCUA__UCCCUCCGCC
           _.||______||||__||||||||||
           _UGG______GGAU__AGGGAGGCGG
miRNA:  3' ____UUGCCU____GA__________ 5'
```

Binding Type	UTR position	Score	Conservation	Also predicted by
8mer(pos 1)	853 - 881	1	0	

Position on Chromosome 22: 34278879 - 34278907

Conservation Info Not Conserved

```
3'UTR:  5' _G____UA___AUUGA__________ 3'
           __CAGC__GAC_______CCUCCGCC
           __||.|__|||_______||||||||
           __GUUG__CUG_______GGAGGCGG
miRNA:  3' UG____C____GAUGAAG________ 5'
```

4	hsa-miR-124	8.35	0.56	3.19

PubMed Links: gene | miRNA | both

Binding Type	UTR position	Score	Conservation	Also predicted by
7mer(pos 2)	8 - 36	4	5	

5	hsa-miR-940	8.00	0.67	3.08	

PubMed Links: gene | miRNA | both

Binding Type	UTR position	Score	Conservation	Also predicted by
7mer(pos 1)	136 - 164	1	1	
8mer(pos 2)	1757 - 1785	1	1	
8mer(pos 2)	1793 - 1821	1	0	

6	hsa-miR-637	8.00	0.43	1.7	

PubMed Links: gene | miRNA | both

Binding Type	UTR position	Score	Conservation	Also predicted by
8mer with wobble(pos 2)	108 - 136	1	0	
7mer(pos 2)	487 - 515	1	0	
8mer(pos 2)	510 - 538	1	1	
7mer(pos 1)	1125 - 1153	1	1	

7	hsa-miR-24	7.30	0.61	2.69	

PubMed Links: gene | miRNA | both

Binding Type	UTR position	Score	Conservation	Also predicted by
7mer(pos 1)	529 - 557	1	0	
8mer(pos 2)	1218 - 1246	1	0	
7mer(pos 2)	1823 - 1851	1	0	

check all | uncheck all | expand selected | collapse selected | expand all | collapse all

B.S.R.C. Alexander Fleming - 34 Fleming Street, 16672, Athens, Greece
