# Supplementary figures and images for: MicroRNA-101 inhibits the expression of Rhes, a striatal-enriched small G-protein, at the post-transcriptional level in vitro
Source: BMC Res Notes. 2018 Jul 31;11:528. doi: 10.1186/s13104-018-3654-5 (PMC6069827; doi:10.1186/s13104-018-3654-5)

## Additional File 2

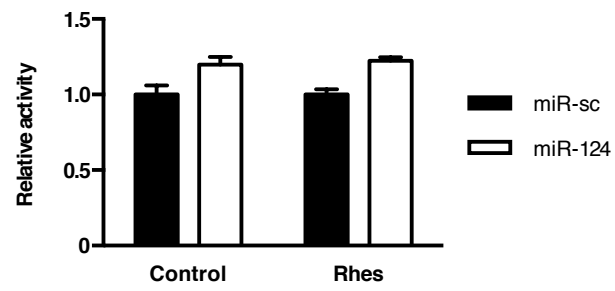

Supplement: Supplementary file 2 — Additional file 2. Luciferase activity was not inhibited by miR-124. Luciferase assay using a portion of the Rhes mRNA 3′UTR and miRNAs. HEK293 cells were co-transfected with a reporter vector without the insert (Control) or with Rhes 3′UTR (Rhes) and 50 nM miRNA mimics (miR-sc or miR-124). At 24 h after transfection, the cells were lysed, and the luciferase activity of the cell lysates was measured. The activity was normalized to that of a control transfected with miR-sc. Data are presented as the mean ± SEM, n = 5. [file 13104_2018_3654_MOESM2_ESM.pdf]
